# A novel animal model of neuropathic corneal pain–the ciliary nerve constriction model

**DOI:** 10.3389/fnins.2023.1265708

**Published:** 2023-12-08

**Authors:** Yashar Seyed-Razavi, Brendan M. Kenyon, Fangfang Qiu, Deshea L. Harris, Pedram Hamrah

**Affiliations:** ^1^Center for Translational Ocular Immunology, Tufts Medical Center, Boston, MA, United States; ^2^Department of Ophthalmology, Tufts Medical Center, Tufts University School of Medicine, Boston, MA, United States; ^3^Program in Neuroscience, Graduate School of Biomedical Sciences, Tufts University, Boston, MA, United States; ^4^Departments of Neuroscience and Immunology, Tufts University School of Medicine, Boston, MA, United States

**Keywords:** neuropathic corneal pain, ocular pain, nociception, chronic constriction injury, animal model

## Abstract

**Introduction:**

Neuropathic pain arises as a result of peripheral nerve injury or altered pain processing within the central nervous system. When this phenomenon affects the cornea, it is referred to as neuropathic corneal pain (NCP), resulting in pain, hyperalgesia, burning, and photoallodynia, severely affecting patients’ quality of life. To date there is no suitable animal model for the study of NCP. Herein, we developed an NCP model by constriction of the long ciliary nerves innervating the eye.

**Methods:**

Mice underwent ciliary nerve constriction (CNC) or sham procedures. Safety was determined by corneal fluorescein staining to assess ocular surface damage, whereas Cochet-Bonnet esthesiometry and confocal microscopy assessed the function and structure of corneal nerves, respectively. Efficacy was assessed by paw wipe responses within 30 seconds of applying hyperosmolar (5M) saline at Days 3, 7, 10, and 14 post-constriction. Additionally, behavior was assessed in an open field test (OFT) at Days 7, 14, and 21.

**Results:**

CNC resulted in significantly increased response to hyperosmolar saline between groups (*p* < 0.0001), demonstrating hyperalgesia and induction of neuropathic pain. Further, animals that underwent CNC had increased anxiety-like behavior in an open field test compared to controls at the 14- and 21-Day time-points (*p* < 0.05). In contrast, CNC did not result in increased corneal fluorescein staining or decreased sensation as compared to sham controls (*p* > 0.05). Additionally, confocal microscopy of corneal whole-mounts revealed that constriction resulted in only a slight reduction in corneal nerve density (*p* < 0.05), compared to naïve and sham groups.

**Discussion:**

The CNC model induces a pure NCP phenotype and may be a useful model for the study of NCP, recapitulating features of NCP, including hyperalgesia in the absence of ocular surface damage, and anxiety-like behavior.

## 1 Introduction

Neuropathic corneal pain (NCP) is a form of neuropathic pain that affects the cornea (Belmonte et al., 2017; Dieckmann et al., 2017). This results in neurosensory abnormalities, manifesting as symptoms such as pain, discomfort, burning, grittiness, irritation, photoallodynia, and severe dryness (Smith and Torrance, 2012; Jensen and Finnerup, 2014; Belmonte et al., 2017; Colloca et al., 2017; Dieckmann et al., 2017). Importantly, although NCP has symptoms that overlap with dry eye disease (DED), it is distinguished from DED by a lack of clinical signs or significant discordance of signs and symptoms, and failure to respond to conventional DED treatments (Kovacs et al., 2016; Belmonte et al., 2017; Dieckmann et al., 2017; Bayraktutar et al., 2020; Moein et al., 2020). There is little known about the underlying pathophysiology, and it is difficult both to diagnose and treat, with no FDA approved drugs available (Goyal and Hamrah, 2016; Dieckmann et al., 2017).

NCP has been reported to occur following ocular trauma or surgery, herpes simplex keratitis or herpes zoster ophthalmicus, systemic autoimmune diseases, as well as in patients with small fiber neuropathy, among others, and results in hyperalgesia and allodynia (Bayraktutar et al., 2020; Yavuz Saricay et al., 2021). Hyperalgesia is a phenomenon by which noxious stimuli elicit a greater pain response than expected, whereas allodynia is a phenomenon by which normally innocuous stimuli now elicit a painful response (Jensen and Finnerup, 2014; Belmonte et al., 2017; Colloca et al., 2017). These neurosensory abnormalities are hallmarks of any neuropathic pain state and are the result of peripheral and central sensitization (Jensen and Finnerup, 2014; Belmonte et al., 2017; Colloca et al., 2017). Among NCP patients, these underlying changes result in symptoms such as pain, burning, photosensitivity, and severe dryness, which have a significant impact on patients’ quality of life (Leonardi et al., 2023).

Nociceptive neurons are sensory neurons specialized for detecting mechanical, thermal, and chemical stimuli. Acute activation of nociceptors by noxious stimuli results in nociceptive pain. Presence of inflammation may result in inflammatory pain (Belmonte et al., 2004a). Neuropathic pain arises from diseases or damage mediated directly to sensory nerves and can result in peripheral sensitization, central sensitization, or both components (Belmonte et al., 2017; Puja et al., 2021). Peripheral sensitization occurs at the nerve terminals of nociceptive neurons. Nerve injury upregulates nociceptive ion channels, enhancing the response to noxious stimuli (Caspani et al., 2009; Del Camino et al., 2010; Lolignier et al., 2016; McKemy, 2018; Pina et al., 2019). These changes are induced by mediators such as inflammatory cytokines, including interleukin (IL)-1β, IL-6, and tumor necrosis factor (TNF)-α (Jensen and Finnerup, 2014; Kovacs et al., 2016; Colloca et al., 2017; McKemy, 2018; Guerrero-Moreno et al., 2020). Central sensitization occurs within the central nervous system at second order neurons. In NCP, the sensory and nociceptive fibers originate from the trigeminal ganglion and second order neurons reside within the brainstem at the trigeminal nucleus (Bortolami et al., 1991; De Armentia et al., 2000; Meng and Kurose, 2013; He and Bazan, 2016). Ultimately, synaptic plasticity enhances the responsiveness of second order neurons, not only to nociceptive inputs but also to non-nociceptive inputs, resulting in allodynia (Jensen and Finnerup, 2014; Colloca et al., 2017).

Although certain features of NCP and DED overlap, the two have a distinct underlying pathophysiology (Belmonte et al., 2017; Dieckmann et al., 2017; Ong et al., 2018; White et al., 2020). Whereas there are several DED models (Yeh et al., 2003; Barabino et al., 2005; Stevenson et al., 2014), these are inappropriate for the study of NCP, given that they result in ocular surface damage and nociceptive pain, but not in neuropathic pain. An alkali burn model exists (Bai et al., 2016; Xiang et al., 2017; Cho et al., 2019); however, this is a harsh model and relevant to only a minor subset of NCP patients. Further, this model results in concurrent nociceptive, inflammatory, and neuropathic pain. These additional factors make the utility of this model for the study of NCP challenging. While there are multiple etiologies that can cause NCP, to further our understanding of NCP’s pathophysiology, an NCP model should eliminate, or at least mitigate, the contributions of these confounders. Thus, there currently is no satisfactory model of NCP and there is an urgent need for a preclinical NCP model to further understanding of the disease and develop new treatment strategies. This is essential as standard treatments alone do not provide significant benefit to NCP patients (Dieckmann et al., 2017).

The cornea receives sensory innervation from the long ciliary nerves, which themselves are branches off the ophthalmic division of the trigeminal nerve (Belmonte et al., 2004a,b; Al-Aqaba et al., 2010). Thus, we hypothesized that corneal nerve dysfunction may be induced by constriction of the long ciliary nerves. Herein, we describe a novel ciliary nerve constriction (CNC) approach and comprehensively characterize changes to corneal nerve function and behavior indicative of an NCP phenotype.

## 2 Materials and methods

### 2.1 Animals

C57BL/6 N male mice 8–10 weeks of age were purchased from Charles River Laboratories (Charles River Laboratories, Shrewsbury, MA) and used in all experiments. Mice were housed in the Tufts Comparative Medicine Services animal facility and kept on a 12/12 light–dark cycle with access to food and water *ad libitum*. Mice were treated in accordance with the Association for Research in Vision and Ophthalmology’s statement on the Use of Animals in Vision and Ophthalmology Research. Additionally, all studies were performed with the approval of the Institutional Animal Care and Use Committee at Tufts University and Tufts Medical Center, Boston, MA (Protocol B2018-47).

### 2.2 Ciliary nerve constriction and sham surgeries

**Figure 1:**
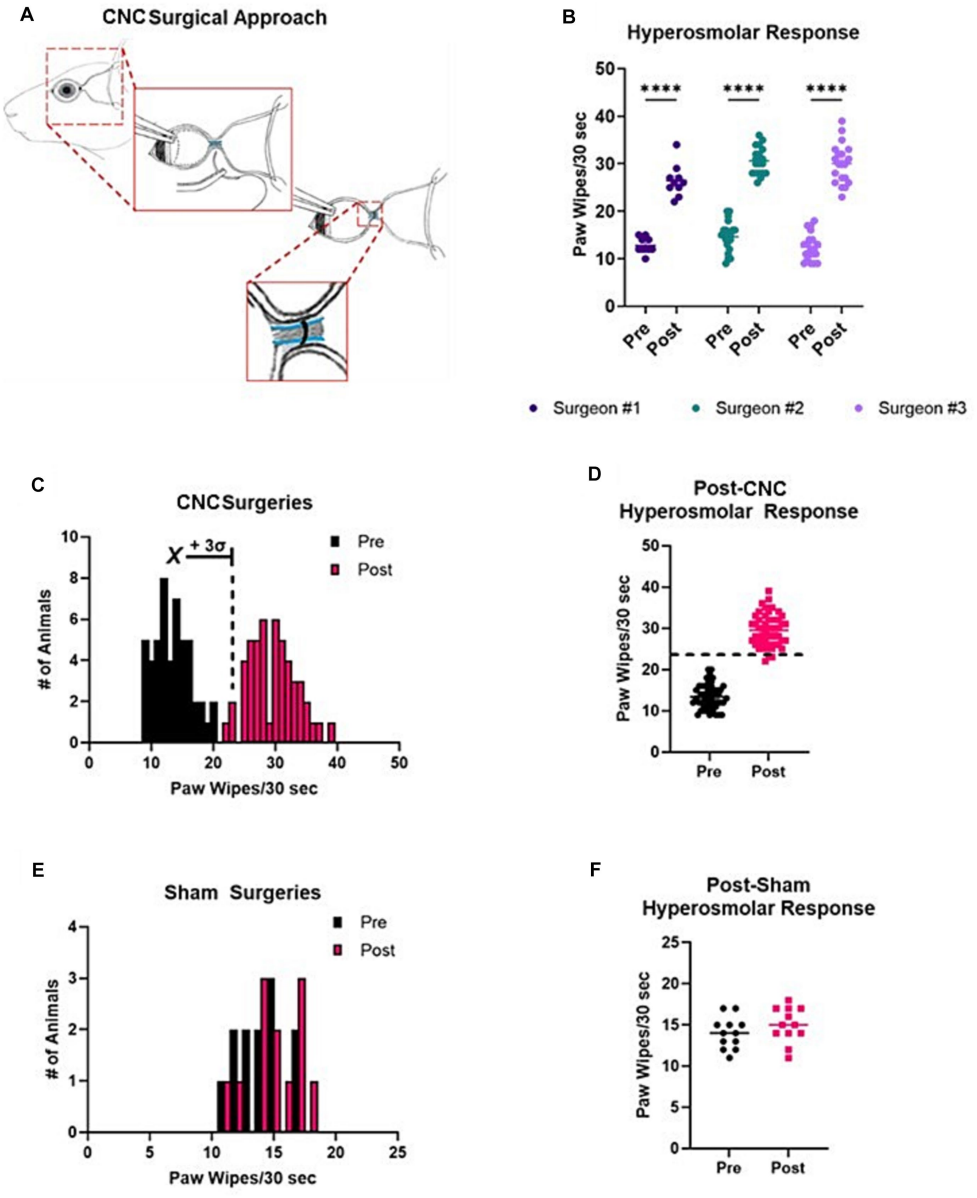
Reproducibility and distribution of responses following CNC or sham surgeries. **(A)** A schematic of the CNC, showing key steps of the procedure. Notice that the ciliary nerves run parallel to the optic nerve. A suture is carefully placed around the ciliary nerves (and optic nerve) with slight tension so as not to sever the ciliary nerves nor damage the optic nerve. **(B)** Pre- and post-CNC hyperosmolar responses across three different surgeons (*n* = 10, 20, and 20, respectively). **(C)** A histogram view of all pre- and post-CNC hyperosmolar responses from **A** reveals a normal distribution of data in both settings. The mean of baseline (pre) responses +3 standard deviations is indicated. **(D)** The same data shown as a scatter plot allows for visualization of the cut-off value for a successful surgery. **(E)** Pre- and post-sham surgery hyperosmolar responses, indicating a normal distribution of the data. **(F)** The same data from **E** shown as a scatter plot demonstrates no difference in pre- and post-sham surgery responses.

Animals underwent unilateral surgery for induction of pain. The long ciliary nerves were injured via chronic constriction, akin to the sciatic nerve chronic constriction injury model, following exposure through a lateral conjunctival approach as previously described (Yamaguchi et al., 2013). A diagrammatic overview of the surgical procedure is denoted in Figure 1A and an overview of the study design is provided in Supplementary Figure S1. In brief, the fur around the lateral aspect of the eye was carefully removed with a razor blade, and the exposed skin sterilized using povidone-iodine and 70% ethanol in deeply anesthetized [Ketamine (100 mg/kg; Zoetis Inc., Kalamazoo, MI) and Xylazine (40 mg/kg; Akorn, Lake Forest, IL)] animals. Following application of topical anesthetic (0.5% proparacaine; Bausch and Lomb, Bridgewater, NJ), a small (~2 mm) incision was made into the lateral canthus and two 6–0 nylon tractional sutures (MicroSurgical Technology, Redmond, WA) placed, the globe rotated nasally, and the lateral conjunctiva was carefully dissected to expose the posterior sclera and optic nerve. The long ciliary nerves run parallel along the optic nerve as they enter the posterior globe. An 8–0 silk suture (Surgical Specialties, Westwood, MA) was carefully placed around the optic nerve and long ciliary nerves. The constriction was placed such that the tension was sufficient to cause dysfunction in the ciliary nerves without severing them and damaging the optic nerve. After constriction, a topical antibiotic was applied to the ocular surface and incision site, a complete tarsorrhaphy was performed using 6–0 nylon sutures (MicroSurgical Technology), and the incision site was closed with 6–0 nylon sutures. Additionally, the mice were administered a single subcutaneous dose of slow-release buprenorphine (1 mg/kg, ZooPharm, Laramie, WY) for post-operative analgesia. On post-operative Day 3, the tarsorrhaphy sutures were carefully removed under an inhaled anesthetic (2% isoflurane). Controls for experiments include sham surgery (animals undergoing all steps of the surgical procedure except for the constriction) and naïve controls (animals which had no surgical procedure performed). A naïve group was included to highlight the natural response of naïve animals. Differences between mice undergoing ligation or sham surgery were then investigated. Contralateral eyes were not used for controls. Baseline in the study design (Supplementary Figure S1) refers to the response of an animal prior to undergoing a CNC or sham surgery. The same animals were tracked over time and assessed throughout each set of experiments for clinical and behavioral outcomes (generally, Sham = 12 and CNC = 30 unless otherwise indicated in respective figure legends). Additional cohorts were used to assess corneal innervation (n = 5/group) and changes in the trigeminal ganglia via qRT-PCR (n = 6/group).

### 2.3 Palpebral opening

Palpebral opening as a measure of spontaneous ocular pain in mice was assessed as previously described (Fakih et al., 2019; Mecum et al., 2020; Fakih et al., 2021). In brief, animals were placed on a 10 cm × 10 cm × 7.5 cm platform and acclimated to the platform for 5 min, after which video recording (Samsung Galaxy S20; Samsung Electronics, Suwon-si, South Korea) of animals began and continued for another 5 min. The video files were screened, in a blinded manner, for frames that would be appropriate for making the measurements. For a frame to be appropriate, the eye of interest must be perpendicular to the axis of the camera lens. Three frames per animal were processed in ImageJ to measure the distance between upper and lower lids (*y*) and nasal and temporal canthi (*x*); the *y*/*x* ratio gives an indication of how open or closed the lids are. Spontaneous pain results in the lids being more closed to limit exposure of the ocular surface, and thus, a decrease in the *y*/*x* ratio. Due to the presence of tarsorrhaphy sutures at Day 3, palpebral opening was assessed at Day 7.

### 2.4 Corneal nociceptive responses

To assess the nociceptive responses of corneal nerves, animals were challenged with 10 μL of hyperosmolar [5 M] saline as previously described (Farazifard et al., 2005; Cho et al., 2019), with a greater paw wipe response being an indicator of an increase in pain. The number of paw wipes were counted in the ensuing 30-s interval, after which the ocular surface was irrigated with normal saline. The experimenter was blinded to the subject’s group. Nociceptive responses were assessed at Days 3, 7, 10, and 14 – these time points were selected based on similar studies (Farazifard et al., 2005) and to minimize any adverse effect of chronic hyperosmolar saline exposure.

### 2.5 Open field test

Behavioral testing was conducted at the Tufts University Circuits & Behavior Core. Animal behavior was assessed in an open field arena for a duration of 10 min per session. The arena consists of a 40 cm × 40 cm open field and is outfitted with a photobeam frame for determination of photobeam breaks. Raw data were analyzed by an automated process using MotorMonitor (KinderScientific, Chula Vista, CA). Parameters used for the analysis included the following: total distance, distance in center, distance in periphery, immobility, entries into the center, and entries into the periphery. The center was defined as the 20 cm × 20 cm central area, the rest was defined as the periphery. Total distance (cm) represents the total distance traveled by the animal, which can be broken down into the distance traveled in the center (cm) and periphery (cm). Entries into the central (counts) and peripheral (counts) regions are also determined, as well as immobility (sec) which is the time in which the animal is not moving. These outcomes were assessed at Days 7, 14, and 21 as behavioral changes may take longer to develop than the pain phenotype.

### 2.6 Mice-nesting consolidation behavior

As a measure of resting behavioral change following CNC, the degree of nesting consolidation was graded on a scale ranging of 1–5, as previously described (Oliver et al., 2018). In short, mice were placed in clean animal cages with cotton nesting material placed on opposing sides, and the degree of consolidation was scored after 5 h. Animals were housed in social groups according to their surgical procedure (constriction or sham). Scoring was as follows: 1–no consolidation; 2–two pieces of cotton nesting grouped; 3–three pieces of cotton nesting grouped; 4–all cotton nesting grouped; and 5–all cotton nesting grouped and shredded (Oliver et al., 2018). Animals were assessed at 7 Days post-CNC or sham surgery.

### 2.7 Gene expression in trigeminal ganglia

At the termination of experiments on Day 14, trigeminal ganglia (TGs) were collected, and RNA extracted for downstream analyses by RT-qPCR. TGs were chopped into small pieces and digested to generate a single cell suspension. Digestion was performed using an enzyme mix of 2 mg/mL Collagenase D, 2 mg/mL DNase I, and 5 mg/mL Dispase II for 30 min at 37°C. The samples were then filtered through a 70 μm cell strainer and the enzymatic digest stopped with DMEM media containing 10% FBS. The cell pellet was then stored in RNALater at −20°C until processing for RNA isolation. RNA isolation was performed using the Qiagen RNeasy Mini Kit. Isolated RNA was reverse-transcribed into cDNA using the iScript cDNA Synthesis Kit (Bio-Rad, Hercules, CA), pre-amplified using the Pre-Amplification Master Mix (Bio-Rad), and SYBR green (Bio-Rad) was used in the RT-qPCR reactions. Primer sequences appear in the (Table 1).

**Table 1:** Primer sequences used in RT-qPCR reactions.

Gene	Forward Primer (5′➔3′)	Reverse Primer (5′➔3′)	Reference
IL-1β	CAACCAACAAGTGATATTCTCCATG	GATCCACACTCTCCAGCTGCA	Li et al. (2011)
IL-6	GAGGATACCACTCCCAACAGACC	AAGTGCATCATCGTTGTTCATACA	Li et al. (2011)
TNFα	CACGTCGTAGCAAACCACCAAGTGGA	TGGGAGTAGACAAGGTACAACCC	Li et al. (2011)
P2X4	CCAACACTTCTCAGCTTGGAT	TGGTCATGATGAAGAGGGAGT	Lalo et al. (2008)
P2X7	GGGGGTTTACCCCTACTGTAA	GCTCGTCGACAAAGGACAC	Lalo et al. (2008)
NGF	AGCATTCCCTTGACACAG	GGTCTACAGTGATGTTGC	Wyatt et al. (2011)
BDNF	TGCAGGGGCATAGACAAAAGG	CTTATGAATCGCCAGCCAATTCTC	Huang and Krimm (2010)
GDNF	TTATGGGATGTCGTGGCTGT	ACACCGTTTAGCGGAATGCT	Mwangi et al. (2010)
NT-3	CATGTCGACGTCCCTGGAAATAG	GGATGCCACGGAGATAAGCAA	Yang et al. (2012)
GAPDH	CCCACTAACATCAAATGGGG	GATGATGACCCTTTTGGCTC	Jamali et al. (2020)

### 2.8 Corneal fluorescein staining

To determine the integrity of the corneal epithelium, corneal fluorescein staining was conducted and scored according to the National Eye Institute (NEI) Scale (Lemp, 1995). This was done to ensure that the ocular surface was not damaged by the surgery. Fluorescein solution was prepared by reconstituting 1 mg of fluorescein from a FUL-GLO Fluorescein Strip (Akorn, Lake Forest, IL) in 1 mL of 1X phosphate buffered saline (PBS; Gibco, Billings, MT) resulting in a 0.1% fluorescein solution. Briefly, animals were anesthetized with Ketamine (100 mg/kg) and Xylazine (40 mg/kg), and a 0.1% fluorescein solution was instilled to the ocular surface for 30 s; after which the solution as removed with a cotton-tip applicator and the ocular surface was rinsed with normal saline three times. The ocular surface was examined by slit-lamp microscopy (SL-1E Model, TopCon, Oakland, NJ) and CFS graded from 0 to 3 in the central cornea and four peripheral regions by NEI scale (Lemp, 1995). This scale is used to quantify the amount and distribution of staining, ranging from 0 (no staining) to 3 (significant staining). The score from each of the 5 regions is combined to give a total score out of 15.

### 2.9 Cochet-Bonnet esthesiometry

To assess the integrity of corneal sensation following surgical procedures, Cochet-Bonnet esthesiometry was conducted (Chao et al., 2015). Briefly, animals were restrained and the Cochet-Bonnet esthesiometer (Western Ophthalmics, Lynnwood, WA) was touched to the central cornea starting at the maximum length of 6 cm. A positive response was recorded when the animal had a blink reflex in response to at least 2 out of 3 challenges with the monofilament. If the animal did not respond (absence of blink reflex), the length of the monofilament was decreased by 0.5 cm and the animal rechallenged. The length of the monofilament inversely correlates with the pressure being applied, thus the longer the length then the less pressure applied. For clarity, the values of length (cm) obtained by Cochet-Bonnet esthesiometry were converted into pressure (g/mm^2^).

### 2.10 Immunofluorescent staining

Corneas were collected from some animals at Days 14 and 28 to assess corneal innervation. Day 14 was selected to as it coincides with observed behavioral changes, and Day 28 was selected to look for any late changes that may occur. Corneas were excised and fixed for immunofluorescent staining. The corneas were fixed in 100% cold acetone (Sigma-Aldrich, St. Louis, MO) for 20 min, washed three times in PBS, and blocked in 3% bovine serum albumin (BSA; Sigma-Aldrich) overnight at 4°C. The corneas were then stained with a pan-neuronal anti-β_III_-tubulin NorthernLights557-conjugated antibody (1:100 dilution; R&D Systems, Minneapolis, MN) overnight at 4°C, after which corneas were washed three times in PBS and mounted using DAPI mounting medium (Vectashield Mounting Medium with DAPI; Vector Laboratories, Burlingame, CA). Whole-mounted corneas were imaged using a Nikon A1R Confocal Microscope (Nikon Healthcare, Tokyo, Japan; Tufts Center for Neuroscience Research–Cell Imaging and Analysis Core), and the NorthernLights557 was excited using the 561 nm laser.

### 2.11 Nerve density analysis

Acquired images were analyzed in ImageJ using the NeuronJ plugin for nerve tracing analysis, as previously described (Yamaguchi et al., 2013). Briefly, z-stacks were converted into maximum projection intensity for subbasal and stromal nerves. The maximum projections were converted to 8-bit black and white images and then manually traced using the NeuronJ plugin. Raw values were then converted into mm/mm^2^, to account for the conversion of pixels to microns and the scan area (Yamaguchi et al., 2013).

### 2.12 Statistical Analyses

For all studies, mice were randomized into sham or CNC groups. Statistical Analyses were conducted in GraphPad Prism v9.4.1 (GraphPad, San Diego, CA). Normality of the data was assessed using the Shapiro–Wilk test. For normally distributed data, the comparison between two groups, a Student’s t-test was used; comparisons of three or more groups were conducted using a One-Way ANOVA with Bonferroni’s post-hoc comparison test was used. For any non-normally distributed data, the Kruskal-Wallis Test was used. Analyses were considered statistically significant with a value of *p* <0.05. Data are presented as Mean ± SEM, unless otherwise indicated.

## 3 Results

### 3.1 Ciliary nerve constriction is a reproducible surgical approach for induction of neuropathic corneal pain

In an effort to establish a reproducible surgical model, three different investigators with varying degrees of surgical experience, were instructed on the surgical approach for our CNC model. Evoked pain responses to hyperosmolar saline were assessed at baseline and 3 Days post-CNC. The results of these are shown in Figure 1B. Responses were increased post-CNC for all three surgeons. Combining the data from all three surgeons allowed for determination of a cut-off value to determine if a surgery was successful. In Figure 1C, both pre- and post-CNC responses have a normal distribution of data, as determined by Shapiro-Wilke normality test (*p* > 0.05). Thus, taking the mean (13.40) + 3 standard deviations (2.94) of the pre-CNC responses gives an upper limit for pre-CNC hyperosmolar responses of 22.22 paw wipes/30 s. Therefore, responses above this value are likely to come from a different population, i.e., animals with an NCP phenotype. Thus, to be even more stringent, we determined that for a surgery to be successful, the post-CNC response must be >24 paw wipes/30 s, as indicated by the dashed line in Figure 1D. Furthermore, to ensure that the observed phenotype was due to the constriction itself and not the other steps of the surgical approach, a subset of animals underwent sham surgeries. As indicated in Figures 1E,F, the pre- and post-sham surgery responses overlap completely with each other. Taken together, this confirms that the CNC model is reproducible and that the NCP phenotype is only observed in CNC but not sham animals.

### 3.2 Ciliary nerve constriction results in altered spontaneous and evoked discomfort or pain response

**Figure 2:**
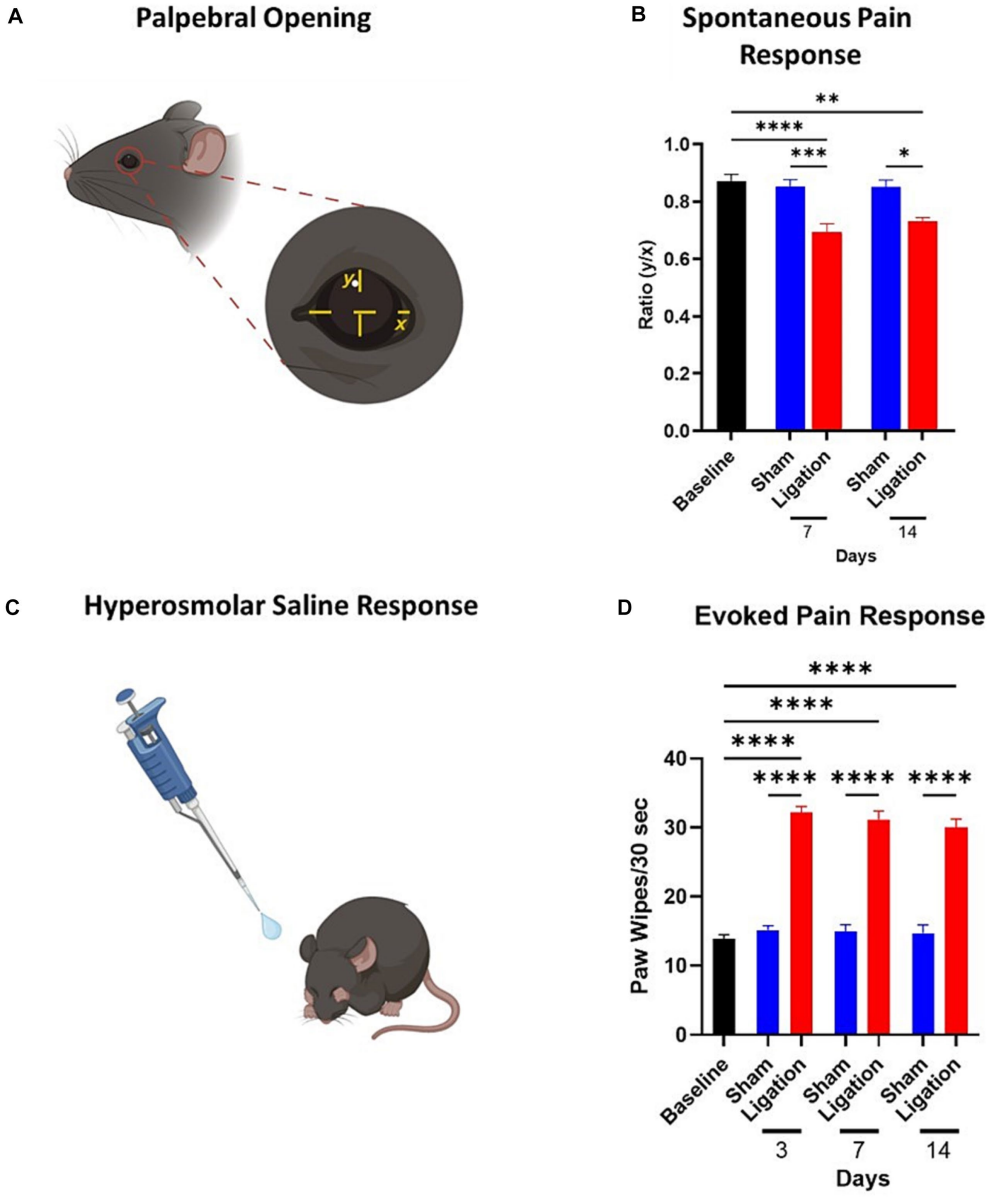
Assessment of spontaneous and evoked pain responses. **(A)** Depiction of the measurements taken for calculation of palpebral opening ratios, an indicator of spontaneous pain. **(B)** Quantification of palpebral opening ratios as a marker of spontaneous pain at baseline and Days 7 and 14 (*n* = 9/group). **(C)** Depiction of hyperosmolar saline response, resulting in characteristic paw wipe response to this stimulus. **(D)** Quantification of the paw wipes as a marker of an evoked pain response to application of hyperosmolar saline. Note the pronounced increase in the CNC animals from Day 3 post-CNC until termination of the study (Sham: *n* = 11; CNC: *n* = 19). Statistical analysis: One-Way ANOVA; *, *p* < 0.05; **, *p* < 0.01; ***, *p* < 0.001; ****, *p* < 0.0001. Panels **A,C** were created with BioRender.

We assessed the palpebral opening (Figure 2A) of animals following CNC, which has been used as an indicator of spontaneous ocular pain (Fakih et al., 2019, 2021). We observed a significant decrease in palpebral opening in CNC animals, corresponding to spontaneous pain at Days 7 (CNC: 0.69 ± 0.08 vs. sham: 0.85 ± 0.03, *p* = 0.0004) and 14 (CNC: 0.73 ± 0.01 vs. sham: 0.85 ± 0.02, *p* = 0.016) post-surgery (Figure 2B). Importantly, the palpebral opening of animals in the sham groups were comparable to pre-surgical baseline values (0.87 ± 0.02, *p* > 0.05; Figure 2B), whereas CNC animals had a decreased ratio at Days 7 and 14 compared to baseline (*p* < 0.0001 and *p* = 0.0021, respectively).

Corneal nociceptors can respond to a variety of stimuli, including warming, cooling, osmolarity, dryness, chemical irritation, and mechanical stimulation, and are classified by their response to these stimuli (Carr et al., 2003; Alamri et al., 2018; Guerrero-Moreno et al., 2020). Thus, to confirm nociceptor hypersensitivity, mice were challenged with hyperosmolar saline (Figure 2C). As expected, responses from the sham group did not differ from baseline (13.86 ± 0.62 wipes) at any timepoint, whereas the CNC animals had significantly increased responses at all timepoints. Furthermore, the hyperosmolar saline challenge resulted in a pronounced increase in the pain response in CNC animals compared to sham controls, as indicated by a significant increase in the paw wipe response at all timepoints assessed, including Day 3 (15.18 ± 0.60 vs. 32.21 ± 0.88 wipes, *p* < 0.0001); Day 7 (14.90 ± 1.06 vs. 31.17 ± 1.24 wipes, p < 0.0001); and Day 14 (14.70 ± 1.21 vs. 30.05 ± 1.19 wipes, *p* < 0.0001; Figure 2D).

### 3.3 Ciliary nerve constriction results in anxiety-like behavior

To further assess this model as inducing a pain phenotype, the CNC and sham animals were assessed for behavioral changes with mice-nesting consolidation (NC) and open field (OF) testing. The rationale for the extended time course for our behavioral analyses (as well as for nerve density) was due to alterations to the central nervous system, required for anxiety behavior, can take longer to present. Alterations in animal activity was assessed using the mice-nesting consolidation test (Oliver et al., 2018) where the degree of nesting over a period forms an indication of activity. A lower NC score reflects less activity and thus, presumably, increased pain. CNC mice showed significantly less consolidation of cotton nesting material compared to mice undergoing a sham procedure (Supplementary Figures S2A–C; scoring: 2.75 ± 0.25 vs. 4.75 ± 0.25, *p* = 0.0013).

**Figure 3:**
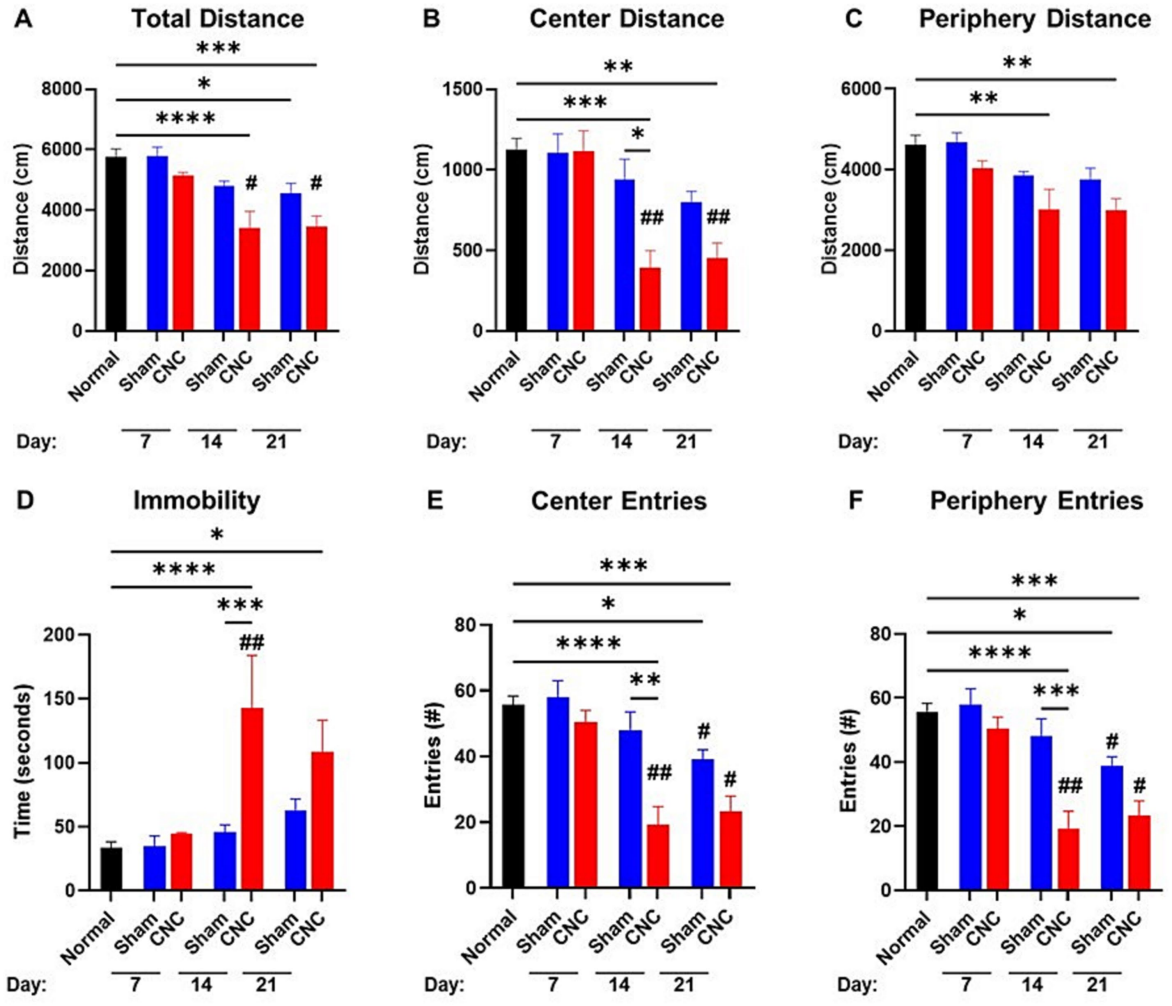
Open field test shows anxiety-like behavior following CNC. A 10-min open field test was performed on sham and CNC animals on Days 7, 14, and 21. The following parameters were analyzed: total distance traveled **(A)**, center distance **(B)**, periphery distance **(C)**, immobility **(D)**, center entries **(E)**, and periphery entries **(F)**. Statistical analysis was conducted by a One-Way ANOVA; *p < 0.05; **p < 0.01; ***p < 0.001; and ****p < 0.0001. # denotes significance compared to Day 7 group with #*p* < 0.05 and ## *p* < 0.01. Normal *n* = 10; Sham *n* = 7; CNC *n* = 5.

Analyses of results from the OFT showed the total distance traveled by CNC animals to be significantly decreased at Day 14 compared to naïve controls (CNC vs. naïve: 3,410 ± 543 vs. 5,750 ± 271 cm, *p* < 0.0001), and persisting until Day 21 (3,447 ± 353, *p* < 0.001); although there was a decrease compared to sham animals at all timepoints (CNC vs. sham: 3,410 ± 543 vs. 4,788 ± 171 cm, *p* = 0.0577; and 3,447 ± 353 vs. 4,560 ± 3,328 cm, *p* = 0.2748; Figure 3A), this was not statistically significant. The center distance traveled by CNC animals was significantly decreased by Day 14 compared to normal and sham controls (CNC vs. naïve: 393 ± 106 vs. 1,129 ± 67 cm, *p* = 0.0002; vs. Sham: 939 ± 128 cm, *p* < 0.012; Figure 3B). Furthermore, the periphery distance traveled by CNC animals decreased at Day 14 (CNC vs. naïve: 3,018 ± 493 vs. 4,621 ± 233 cm, *p* = 0.0019) and Day 21 (constriction: 2,993 ± 291 cm, *p* = 0.004), compared to normal controls (Figure 3C). By Day 14, CNC animals had a significantly higher amount of time spent immobile compared to normal controls (constriction vs. control: 143.0 ± 41.0 vs. 33.6 ± 4.4 s, *p* < 0.0001) and sham controls (sham 45.8 ± 5.7 s, *p* = 0.0002; Figure 3D). At Day 14, CNC animals had fewer entries into the center and periphery of the arena, indicating a decrease in exploratory behavior and heightened anxiety following CNC (Figures 3E,F). While mice undergoing sham surgery had a slight decline in total distance at Day 21 (sham vs. control: 4,560 ± 323 vs. 5,750 ± 271 cm, *p* = 0.0437), no significant difference was noted when assessing distance traveled in the center (sham vs. control: 800 ± 66 vs. 1,129 ± 67 cm, *p* > 0.05) or periphery (sham vs. control: 3,760 ± 277 vs. 4,621 ± 233 cm, *p* > 0.05), compared to normal controls (Figures 3A–C).

Taken together, these findings suggest that CNC decreases basal activity and increases anxiety-like behavior as assessed by NC and OF testing, respectively, further supporting that pain is induced in this model.

### 3.4 Ciliary nerve constriction results in upregulation of molecular markers of pain in trigeminal ganglia

**Figure 4:**
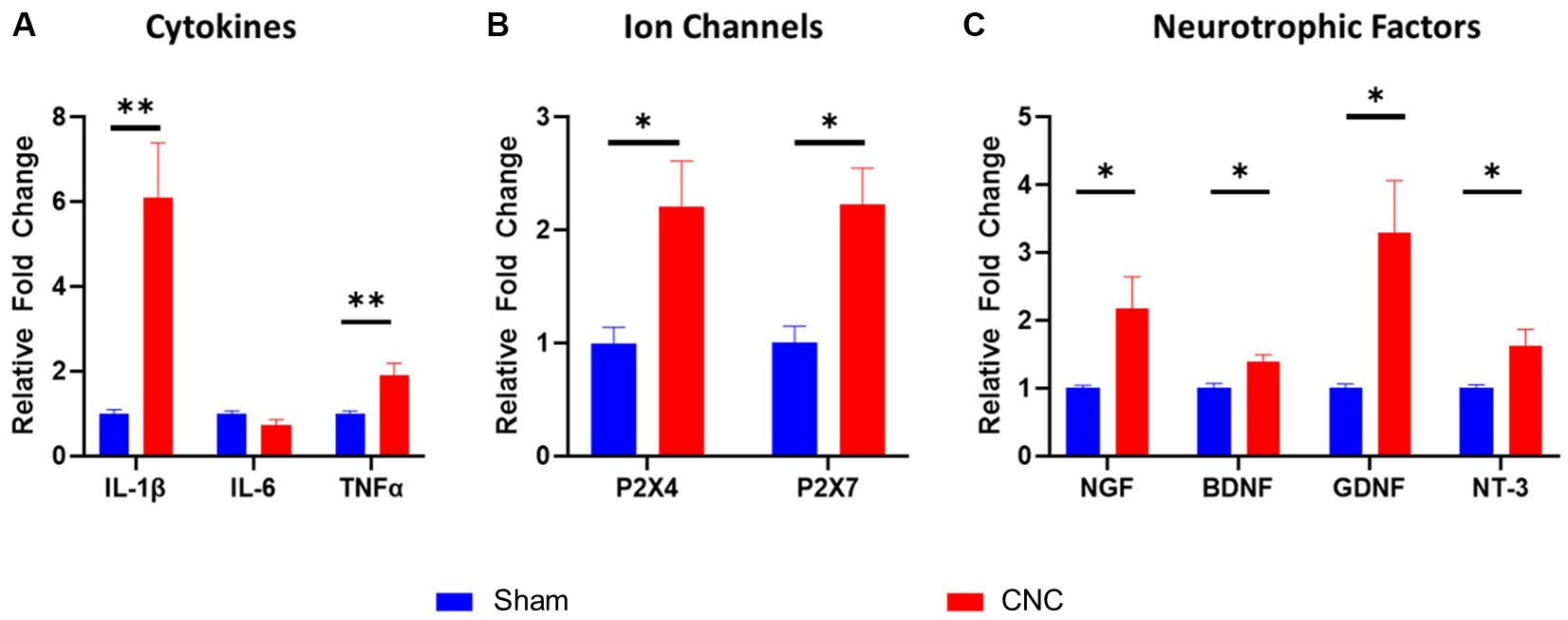
Characterization of molecular alterations in trigeminal ganglia following CNC. The fold change of cytokines **(A)**, ion channels **(B)**, and neurotrophic factors **(C)** were determined by RT-qPCR. Fold change was calculated by the ddCt method and normalized to sham group. Data are presented as means ± SEM. Statistical analysis was conducted using unpaired *t*-test; *, *p* < 0.05; **, *p* < 0.01. *n* = 5-6/group.

RT-qPCR analysis on the ipsilateral trigeminal ganglia of CNC and sham animals was performed to assess cytokines, ion channel and neurotrophic factors. Pro-inflammatory cytokines IL-1β and TNF-α increased in CNC animals compared to sham animals at 6.09 ± 1.31-fold (*p* = 0.002) and 1.90 ± 0.29-fold (*p* = 0.016), although IL-6 did not differ between groups (*p* = 0.108; Figure 4A). Furthermore, while several ion channels are implicated in neuropathic pain, we found that the purinergic ion channels P2X4 and P2X7 were both increased following constriction to 2.32 ± 0.40-fold and 2.22 ± 0.32-fold, respectively (*p* = 0.021 and *p* = 0.0058, respectively; Figure 4B). In our model, the neurotrophic factors NGF (2.18 ± 0.47-fold; *p* = 0.032), BDNF (1.39 ± 0.11-fold; *p* = 0.0012), GDNF (3.29 ± 0.78-fold; *p* = 0.019), and NT-3 (1.63 ± 0.24-fold; *p* = 0.046) were all significantly increased in CNC compared to sham animals (Figure 4C). Taken together, significant upregulation of cytokines, ion channels, and neurotrophic factors was detected in CNC animals, presenting further confirmation of the pain phenotype following CNC.

### 3.5 Characterization of the ocular surface following ciliary nerve constriction reveals minimal damage to the corneal epithelium

**Figure 5:**
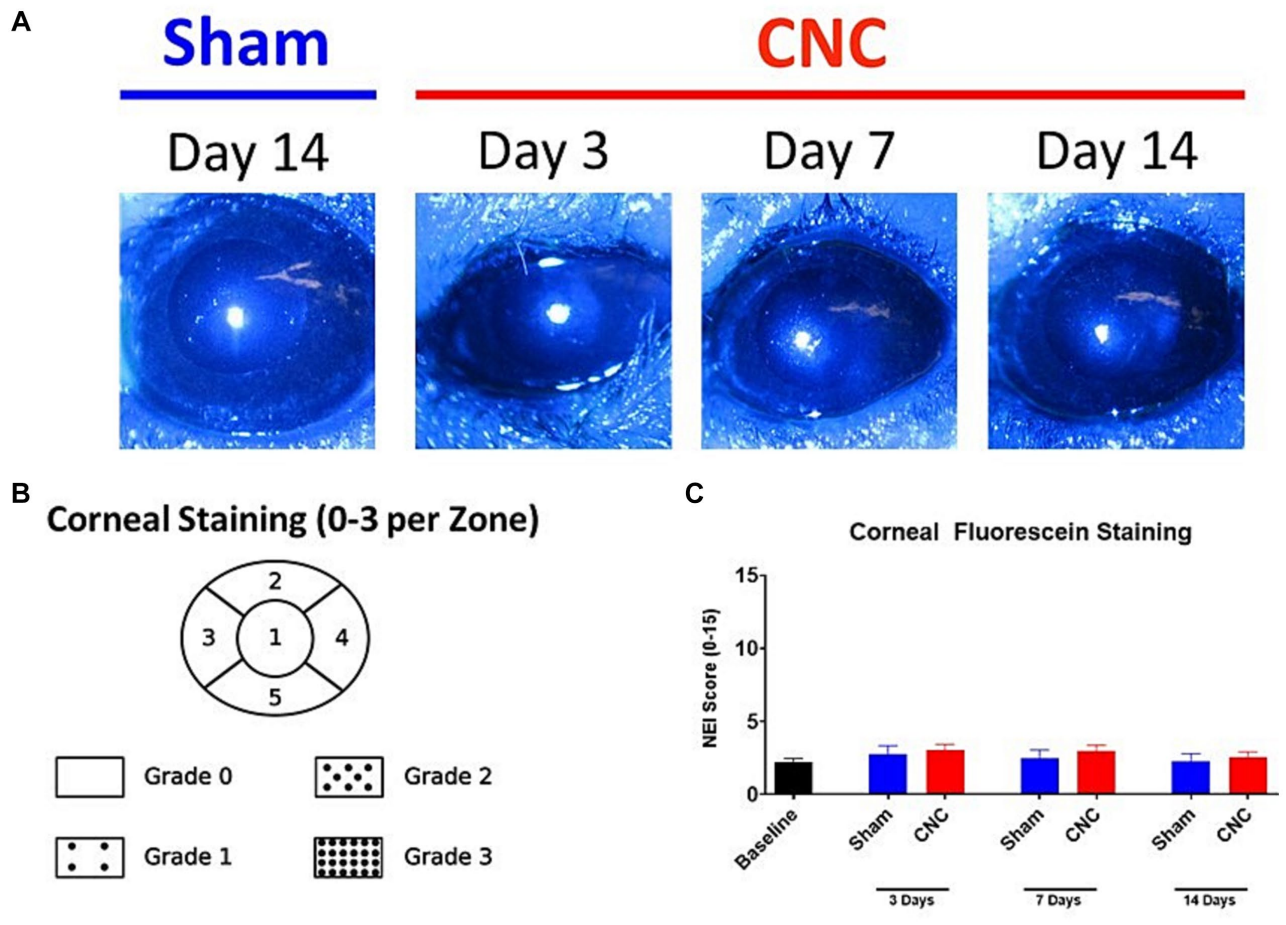
The ocular surface remains undamaged after CNC. **(A)** Representative slit-lamp microscopy images from sham and CNC animals as well as **(B)** quantification of the corneal fluorescein staining according to the NEI scale **(C)**. Note that there is minimal staining of the ocular surface, and there is no significant difference between sham and CNC animals (sham: *n* = 12, CNC: *n* = 30). Statistical analysis: Groups were compared at respective time points by a Kruskal-Wallis test (*p* = 0.4978).

Corneal fluorescein staining (CFS) was conducted following CNC at 3, 7, and 14 Days to determine whether the CNC procedure had any detrimental impact on the ocular surface, specifically the corneal epithelium. Minimal staining of the corneal epithelium was noted following CNC (see Figure 5A for representative slit-lamp microscopy images). Scoring by the NEI scale (Figure 5B) revealed no significant differences in scores between sham and CNC animals at Days 3 (CNC vs. sham: 3.03 ± 0.41 vs. 2.75 ± 0.59, *p* > 0.9999), 7 (CNC vs. sham: 3.00 ± 0.37 vs. 2.5 ± 0.54, *p* > 0.9999), and 14 post-surgery (CNC vs. sham: 2.57 ± 0.34 vs. 2.25 ± 0.54, *p* > 0.9999; Figure 5C). Further, no corneal opacity was noted by slit-lamp microscopy at any time point for either group. Importantly, these results indicate that the CNC approach does neither cause overt epithelial defects, nor ocular surface damage as assessed by CFS and slit-lamp microscopy.

### 3.6 Corneal mechanosensation remains intact following ciliary nerve constriction

**Figure 6:**
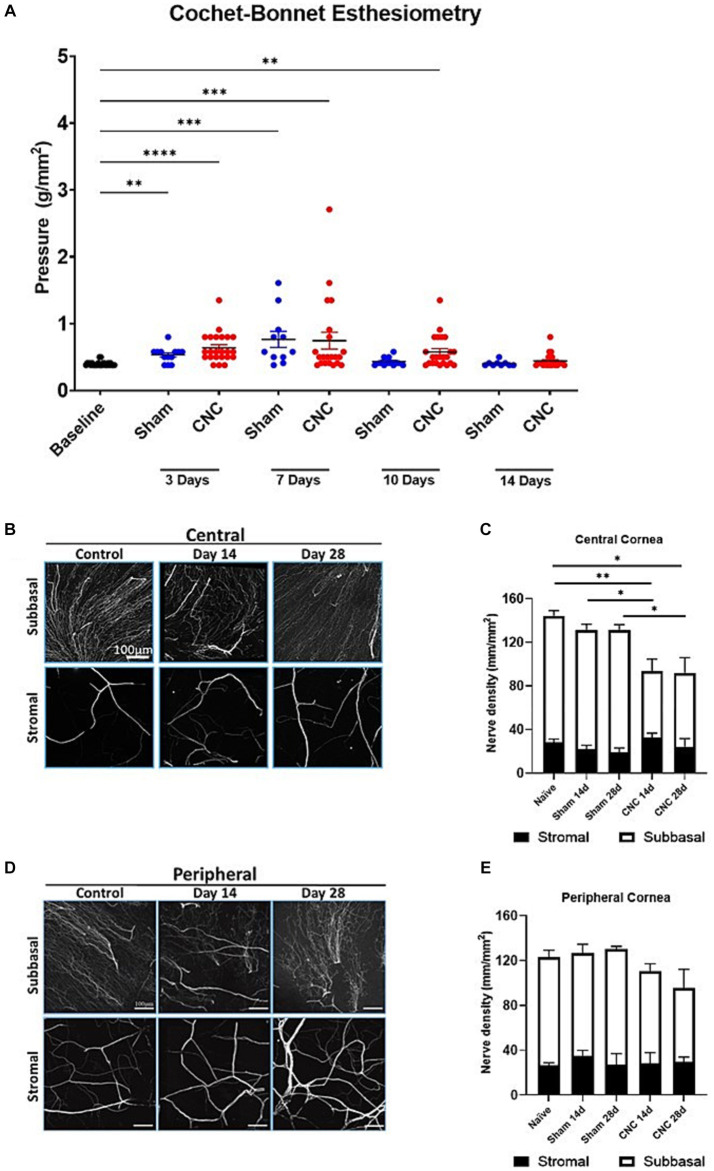
Corneal innervation remains intact following CNC or sham surgery. **(A)** Quantification of corneal mechanosensation as determined by Cochet-Bonnet esthesiometry at Days 3, 7, 10 and 14. **(B)** Representative confocal images of βIII-tubulin staining from corneal whole-mounts in the central cornea – the top row is of the subbasal plexus, and the bottom row is of the stromal nerves. **(C)** Quantification of nerve density in the central cornea – white bars indicate the subbasal plexus and the black bars represent the stromal nerves. **(D)** Representative confocal images of β_III_-tubulin staining from corneal whole-mounts in the peripheral cornea – the top row is of the subbasal plexus, and the bottom row is of the stromal nerves. **(E)** Quantification of nerve density in the central cornea – white bars indicate the subbasal plexus and the black bars represent the stromal nerves. For **A**, Sham = 12 and CNC = 30 and for **B–E** *n* = 5/group. Statistical analysis for **A** was conducted by Kruskal-Wallis test; statistical analysis for **C,E** was conducted using One-Way ANOVA; *p < 0.05; **p < 0.01; ***p < 0.001; ****p < 0.0001.

To ensure innervation remained present following the CNC procedure, Cochet-Bonnet esthesiometry was used to assess response to mechanical stimulus. The blink reflex remained intact in all animals assessed at Days 3, 7, 10 and 14. There were no differences in the pressure needed to elicit a response between CNC and sham groups at Day 3 (CNC vs. Sham: 0.64 ± 0.05 vs. 0.53 ± 0.03 g/mm^2^; *p* > 0.9999), Day 7 (CNC vs. Sham: 0.77 ± 0.12 vs. 0.75 ± 0.13 g/mm^2^; *p* > 0.9999), Day 10 (CNC vs. Sham: 0.43 ± 0.02 vs. 0.58 ± 0.05 g/mm^2^; *p* = 0.7073) and Day 14 (CNC vs. Sham: 0.44 ± 0.02 vs. 0.40 ± 0.01 g/mm^2^; *p* > 0.9999; Figure 6A). Despite this, baseline levels (0.40 ± 0.01 g/mm^2^) for mechanosensation differed from those of the sham group at Days 3 and 7 (*p* = 0.01 and *p* = 0.0001, respectively) and baseline differed from the CNC group at Days 3, 7 and 10 (*p* < 0.0001, *p* = 0.0004, and *p* = 0.0018, respectively). However, our key finding is that the corneal innervation remains intact and responsive to mechanical stimuli throughout the duration of the study.

### 3.7 Constriction has minimal effect on corneal innervation

While our results from Cochet-Bonnet esthesiometry reveal that the corneal innervation remains present, we next assessed the effect of CNC on corneal nerve density by confocal microscopy. Analysis of total nerve density, indicated by staining of a pan-neuronal marker (β_III_-tubulin), revealed a slight, but statistically significant, decrease in the CNC compared to sham or naïve controls at Days 14 and 28 (Figures 6B,C). A transient decline in the central corneal innervation, specifically among the subbasal nerves occurred at Day 14 following constriction (CNC vs. naïve: 73.86 ± 8.81 vs. 116.48 ± 4.70 mm/mm^2^; *p* = 0.0104 and CNC vs. sham: 73.86 ± 8.81 vs. 110.58 ± 7.52 mm/mm^2^; *p* = 0.042) and Day 28 (CNC vs. naïve: 67.54 ± 14.04 vs. 116.48 ± 4.70 mm/mm^2^; *p* = 0.0054 and CNC vs. sham: 67.54 ± 14.04 vs. 111.34 ± 3.61 mm/mm^2^; *p* = 0.0184; Figures 6B,C). Stromal nerves were unaffected at Day 14 (CNC vs. naïve: 30.56 ± 3.27 vs. 28.10 ± 3.14 mm/mm^2^; *p* = 0.9924 and CNC vs. sham: 30.56 ± 3.27 vs. 116.48 ± 4.65 mm/mm^2^; *p* = 0.6184) and Day 28 (CNC vs. naïve: 24.43 ± 7.35 vs. 28.10 ± 3.14 mm/mm^2^; *p* = 0.9734 and CNC vs. sham: 24.43 ± 7.35 vs. 18.17 ± 2.98 mm/mm^2^, *p* = 0.8650; Figures 6B,C). Peripherally, the subbasal plexus did not differ between CNC animals and naïve or sham controls at Day 14 (CNC vs. naïve: 81.22 ± 6.64 vs. 97.60 ± 5.47 mm/mm^2^, *p* = 0.4086; and CNC vs. sham: 81.22 ± 6.64 vs. 98.61 ± 9.18 mm/mm^2^, *p* = 0.4893) or at Day 28 (CNC vs. naïve: 76.95 ± 11.06 vs. 97.60 ± 5.47 mm/mm^2^, *p* = 0.2870; and CNC vs. sham: 76.95 ± 11.06 vs. 101.32 ± 2.43 mm/mm^2^, *p* = 0.2484; Figures 6D,E).

Consistent with the findings in the central cornea, the peripheral stromal nerves did not differ between CNC animals and naïve or sham controls at Day 14 (CNC vs. naïve: 28.79 ± 3.95 vs. 27.40 ± 2.17 mm/mm^2^, *p* = 0.9992; and CNC vs. sham: 28.79 ± 3.95 vs. 30.70 ± 5.57 mm/mm^2^, *p* = 0.9983) or at Day 28 (CNC vs. naïve: 29.57 ± 4.41 vs. 27.402 ± 2.17 mm/mm^2^, *p* = 0.9952; and CNC vs. sham: 29.57 ± 4.41 vs. 28.23 ± 7.09 mm/mm^2^, *p* = 0.9996; Figures 6D,E). Overall, the constriction procedure caused only a slight decrease in corneal innervation, particularly among the central subbasal plexus. However, the rest of the corneal innervation remained unchanged, and taken together with our prior results, suggest that the observed phenotype is the result of underlying nerve dysfunction following constriction.

## 4 Discussion

In the past decade, it has become apparent that NCP represents a distinct pathological condition from DED, although in some patients the two may co-exist (Belmonte et al., 2017; Dieckmann et al., 2017). Importantly, standard treatment for DED seems ineffective for NCP patients (Belmonte et al., 2017; Dieckmann et al., 2017) and there are currently no FDA-approved treatments for NCP. Thus, this necessitates the development of a new model to investigate NCP mechanisms and to test candidate therapeutics. Our results indicate that constriction of the ciliary nerves recapitulates features of NCP, allowing studies on the underlying mechanisms involved in NCP development and progression.

While other ocular pain models do exist–such as the alkali burn model as a model of inflammatory pain (Cho et al., 2019) or various models of DED to study nociceptive pain (Yeh et al., 2003; Barabino et al., 2005; Stevenson et al., 2014) – they are inadequate to study the pathophysiology of NCP. The alkali burn model is well-suited to study the nature of pain following chemical burn (Cho et al., 2019). However, this is relevant for only a fraction of patients, and results in destruction of nerve terminals and damage to the ocular surface. Furthermore, the alkali burn model results in severe inflammation, resulting in combined nociceptive, inflammatory and neuropathic pain. Thus, multiple factors within the alkali burn model contribute to pain, making it less well-suited for the study of NCP in which somatosensory dysfunction predominates (Belmonte et al., 2017). While DED patients may experience pain, this pain is due to the loss of homeostasis at the ocular surface from a decrease in tearing and/or desiccating stress; thus, this would be classified as nociceptive rather than neuropathic pain (Belmonte et al., 2017). Additionally, DED models have confounding factors that negate their utility for the study of NCP. For instance, the scopolamine, which is utilized in a DED model, has direct effects on the nervous system (Birkhimer et al., 1984; Siddik and Fendt, 2022) and the lacrimal gland excision model disrupts the neural circuitry of the ocular surface (Yeh et al., 2003; Viau et al., 2008; Stevenson et al., 2014). Further, neurosensory abnormalities are considered part of dry eye etiology, with chronic dry eye possibly resulting in NCP (Craig et al., 2017). Thus, these models do not closely mimic the pathogenesis of NCP seen clinically, which ultimately is the result of somatosensory nerve dysfunction. Additionally, a recent report from Gao et al. developed a blue light-induced model of ocular pain. Consistent with our own findings, they also observed an increased sensitivity of blue light-exposed animals to hypertonic saline. Interestingly, they also reported an increase in Rose Bengal staining and a decrease in tear secretion (Gao et al., 2023). Thus, although ocular pain is a component of the blue light model, it may more appropriately be considered a DED model with hypersensitivity.

Our murine model of NCP overcomes these obstacles by direct constriction of the ciliary nerves, which provide the sensory innervation to the cornea. Furthermore, this approach does not lead to ocular surface damage as we have shown by assessment of the ocular surface with corneal fluorescein staining, excluding the possibility that this approach induces DED and allowing the pathophysiology of NCP to be studied without confounders. Additionally, our NCP model predominantly results in nerve dysfunction without a pronounced loss of corneal nerves that is seen in other models such as dry eye disease or alkali burn. We observe an increase in the spontaneous pain of CNC animals, as determined by palpebral opening measurements. In addition to spontaneous pain, we also assessed evoked pain in response to hyperosmolar saline and found increased evoked nociceptive responses to topical hyperosmolar saline. The hyperosmolar saline challenge has been used by other groups as an assessment of pain in rodents (Rahman et al., 2014; Cho et al., 2019), which the current results support. Thus, we clearly demonstrate a hypersensitive response of corneal nociceptive neurons in response to hyperosmolar saline following constriction. Together, these findings suggest that corneal nociceptors have been sensitized, which is a dominant feature of neuropathic pain.

In addition, the presence of a neuropathic pain phenotype is further supported by our behavioral studies. Various tests have been utilized to assess changes in pain response, spontaneous pain, and anxiety-like and depression-like behavior (Palazzo et al., 2021). We assessed spontaneous pain (palpebral opening; Akintola et al., 2017; Aulehner et al., 2022), anxiety-like behavior (OF test), and depression-like behavior (nesting behavior; Gallo et al., 2020; Aulehner et al., 2022). We noted a significant difference in the palpebral opening following constriction. Further, as expected of a pain phenotype (Medeiros et al., 2020; Yin et al., 2020; Chen et al., 2021; Goswami et al., 2021), we observed a marked increase in the anxiety-like behavior, most notably at Day 14, and persisting until Day 21, in mice following CNC. Similarly, we noted an increase in depression-like behavior following constriction, as measured by nesting consolidation, compared to sham controls. Of note, it has previously been reported that naïve animals had a nesting consolidation score of 5 (Oliver et al., 2018), which is in line with results of our sham controls. Interestingly, anxiety-like behavior has been reported to be present in mouse models of neuropathic pain prior to depression-like behavior (Yalcin et al., 2011). In our experiments, comparisons between CNC and sham control mice behavior in an OFT did not present significant differences at the earliest timepoint of Day 7, while a difference in nesting consolidation was noted at this timepoint. This difference compared to Yalcin et al.’s findings are likely due to the different behavioral tests used between our work and their own (Yalcin et al., 2011), although this may suggest a difference between trigeminal neuropathic pain and non-trigeminal neuropathic pain. Clinically, it is more challenging to treat trigeminal versus non-trigeminal neuropathic pain, although precise mechanisms for these differences remain elusive (Dieckmann et al., 2017; Obermann, 2019).

Additionally, we investigated molecular markers of pain within the ipsilateral trigeminal ganglia of CNC and sham animals to further validate our NCP model. Consistent with other studies on neuropathic pain, these molecular alterations were assessed at 14 Days post-constriction. This time point was selected as at very early time points the results could be confounded by the stress animals experience from undergoing surgery and from anesthesia, additionally in other neuropathic pain models increases or decreases in gene transcription stabilize between Day 7 and 14 (Renthal et al., 2020). In our model, IL-1β and TNF-α, as well as purinergic ion channels, were significantly increased following constriction. Our results are thus in line with previous neuropathic pain models (Masoodifar et al., 2021; Hu et al., 2022; Luo et al., 2022). These pro-inflammatory cytokines and ion channels have well-established roles in neuropathic pain (Sommer et al., 1998, 1999, 2001; Lindenlaub et al., 2000; Schafers et al., 2001; Ueno et al., 2002). Furthermore, neurotrophic factors were increased following constriction, which has also been described in other models of neuropathic pain (Cirillo et al., 2010; Da Silva et al., 2015, 2017; Lopes et al., 2021; Wu et al., 2021). Thus, these molecular alterations provide further support of a neuropathic pain phenotype.

While there was minimal staining of the cornea by CFS, this was present at baseline, and CNC did not worsen CFS scores. Furthermore, immunofluorescent staining and confocal microscopy revealed only a slight decline in corneal nerve density. However, the results of the Cochet-Bonnet esthesiometry indicate that mechanosensation remains intact with no differences from the sham surgery group. Taken together, these findings suggest that the integrity of the ocular surface is not lost following CNC.

Another surgical constriction model used to induce neuropathic pain in the orofacial area chronic constriction injury of the infraorbital nerve (Vos et al., 1994; Chichorro et al., 2006; Xu et al., 2008; Ding et al., 2017). The infraorbital nerve is a branch of the trigeminal nerve (V) innervating the skin and mucous membranes of the face after traveling through the orbit. Although this method cannot be used as a model for NCP, it can serve as the best comparison for our model in terms of invasiveness and complexity of the method as it is one of few studies utilizing chronic constriction injury / ligation of the trigeminal nerve. Different iterations of infraorbital nerve ligation have been described (Vos et al., 1994; Chichorro et al., 2006; Henry et al., 2015; Ding et al., 2017). These approaches can lead to tissue trauma, damage the facial artery (Ding et al., 2017), as well as taking a long time to correctly perform (over 30 min) (Vos et al., 1994), or are prone to bleeding (Henry et al., 2015). Our model is less complex, taking around 15 min to complete with survival rates of ~95%, and is less invasive as we did not observe any overt damage to the eye.

The current literature indicates inherent differences between the sexes in their responses to pain, pain sensitivity and risk for clinical pain, as well as response to pain interventions (Fillingim et al., 2009; Bartley and Fillingim, 2013). However, assessing the contribution of sex fell outside the scope of the current study, although further experiments are warranted to determine possible sex differences in our CNC model of NCP. Further, it is also not clear what role local inflammation plays following constriction. Thus, further investigation into the underlying mechanisms of CNC is necessary.

In addition to the above, our study has several limitations. These include the possible contribution of central sensitization (Koltzenburg et al., 1994; Ikeda et al., 2012; Baron et al., 2013) to the phenotype observed following CNC. Additionally, assessing behavior in animals is challenging, and there is a continued need for improved tools to accurately assess animal behavior (Oliver et al., 2018; Zhang et al., 2022). Furthermore, the interpretation of behavioral assays has been met with some disagreement in the field or a lack of reproducibility (Gulinello et al., 2019; Tucker and McCabe, 2021). This latter issue may, in part, be due to inherent behavioral differences among different animal strains (Eltokhi et al., 2020). In addition to the role of sex in neuropathic pain, aging may also be a factor–while neuropathic pain may occur at any age, it is more common among older populations (Colloca et al., 2017; Giovannini et al., 2021). However, in the present work mice were all aged 8–10 weeks at the start of the experiment. Furthermore, although our study focused on the cornea, it is possible that other structures innervated by the long ciliary nerves, such as the bulbar conjunctiva, ciliary body, and iris (Forrester et al., 2016), may have been altered by the constriction.

Our CNC approach successfully recapitulates key hallmarks of NCP. This model will enable researchers to investigate the unique molecular and pathophysiologic mechanisms underpinning NCP, for the development of novel therapeutics to treat this debilitating disease.

## Data availability statement

The raw data supporting the conclusions of this article will be made available by the authors, without undue reservation.

## Ethics statement

The animal study was approved by Institutional Animal Care and use Committee at Tufts University and Tufts Medical Center. The study was conducted in accordance with the local legislation and institutional requirements.

## Author contributions

YS-R: Conceptualization, Data curation, Formal analysis, Investigation, Methodology, Writing – original draft, Writing – review & editing. BK: Conceptualization, Data curation, Formal analysis, Investigation, Methodology, Writing – original draft, Writing – review & editing. FQ: Data curation, Formal analysis, Investigation, Writing – review & editing. DH: Data curation, Investigation, Writing – review & editing. PH: Conceptualization, Funding acquisition, Resources, Supervision, Writing – review & editing.
