# Supplementary material for: A novel animal model of neuropathic corneal pain–the ciliary nerve constriction model
Source: Front Neurosci. 2023 Dec 8;17:1265708. doi: 10.3389/fnins.2023.1265708 (PMC10749205; doi:10.3389/fnins.2023.1265708)
Supplement: Supplementary file 1 [file Data_Sheet_1.PDF]

Supplemental Materials

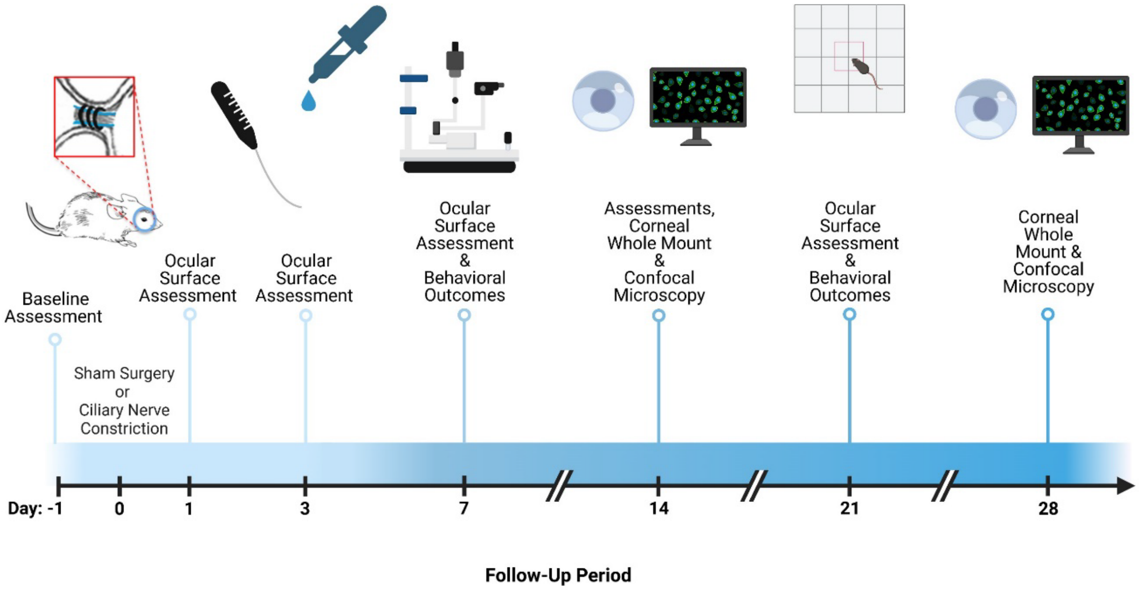

Supplemental Figure 1: Experimental design for the assessment of neuropathic corneal pain. Experimental design for the assessment of pain following CNC. The ocular surface assessments included nociceptive responses to challenge with topical drops, Cochet-Bonnet esthesiometry, and corneal fluorescein staining. Additional outcomes were behavioral assessment in an open field test, and finally assessment of the corneal innervation by confocal microscopy. Created with BioRender.

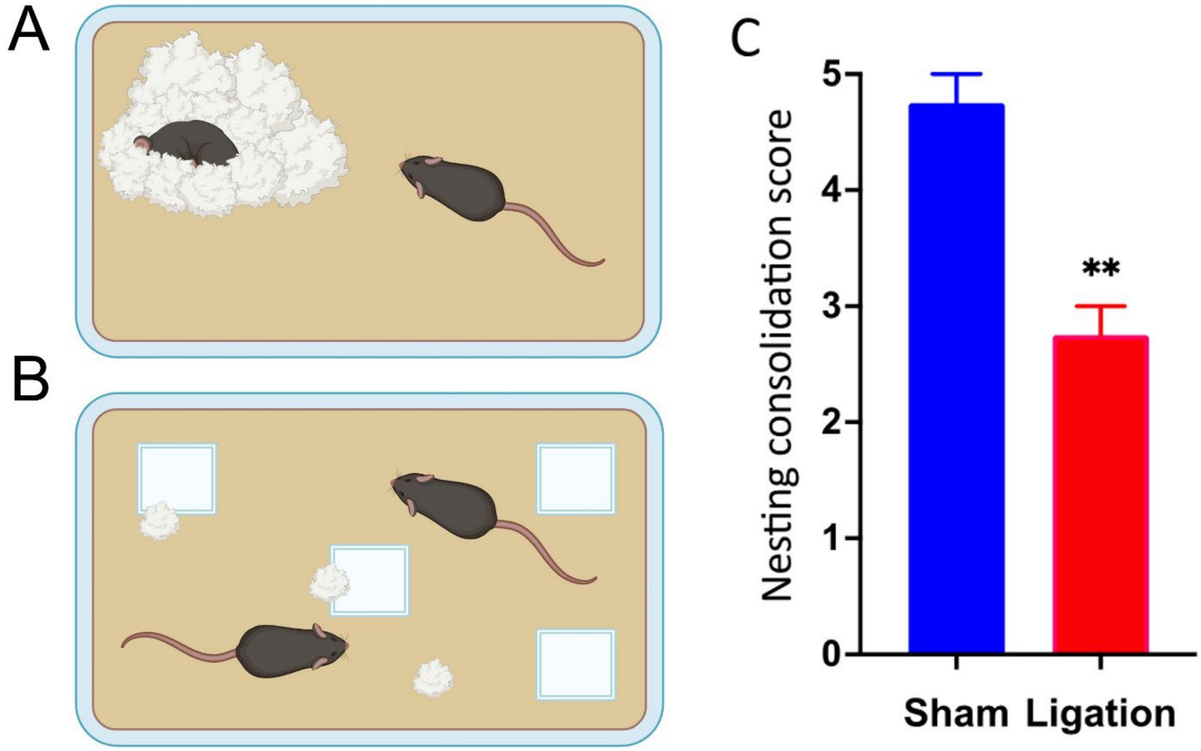

Supplemental Figure 2: Analysis of mice-nesting consolidation as an indication of pain following CNC. Representative images of mice-nesting consolidation of mice that have undergone Sham (**A**) and CNC procedures (**B**). Quantification of cotton nesting material consolidation show altered behavior at 7-day post-surgery (**C**). Statistical analysis was conducted using unpaired t-test; *, $p<0.05$; **, $p<0.01$. Panels **A** and **B** were created with BioRender.
